# What evidence exists on the effect of the main European lowland crop and grassland management practices on biodiversity indicator species groups? A systematic map protocol

**DOI:** 10.1186/s13750-022-00280-0

**Published:** 2022-08-25

**Authors:** Robin Séchaud, Markus Van der Meer, Yvonne Fabian, Philippe Jeanneret

**Affiliations:** Agroecology and Environment, Agroscope, Reckenholzstrasse 191, CH-8046 Zurich, Switzerland

**Keywords:** Agriculture, Bird, Insect, Land-use intensity, Mammal, Pesticide, Plant diversity, Soil biodiversity, Species richness, Species abundance

## Abstract

**Background:**

The intensification of the agricultural practices in Europe over the last decades has drastically transformed the agroecosystems. The simplification of the landscape, the loss of semi-natural habitats and the application of chemicals on crops are known to have led to biodiversity decline in agricultural landscapes, raising substantial concerns about the loss of essential ecosystem services, such as pollination or pest control. Depending on the location, the scale and the regional context, different indicator species groups (ISGs) are often surveyed to assess the state and trend of biodiversity changes in agroecosystems. Although the high diversity of these ISGs allows a broad overview of the biodiversity, it complicates the interpretation of the results and thus their application. In addition, species diversity metrics are various, from simple species counts to more complex measurements of diversity indices, sometimes with antagonistic responses. Here, to meet the pressing need for synthesis in this complex topic, we will follow a standardized systematic map protocol to collect and summarize the literature reporting the effects of the main European lowland agricultural management practices (AMPs) on a set of ISGs.

**Methods:**

Following the systematic evidence synthesis standards, we developed the question to address in the systematic map using the PICO framework. We established a preliminary search string by combining search terms for the categories Population (ISGs), Intervention (AMPs) and Outcome (species diversity), as well as with two additional groups (Environment—to focus on lowland crop and grassland—and Location—to restrict the study area to Europe). We will conduct a comprehensive literature search of relevant peer-reviewed and grey literature using Web of Science and CABI platforms, Google Scholar, specialized websites and through our professional collaborator network. The comprehensiveness of the search will be assessed by comparing the literature collected to a test-list of ninety relevant articles. The repeatability of the literature screening process will be ensured by a list of inclusion/exclusion criteria and inter-reviewer consistency statistical tests. Data extraction will be organized in three complementary tables (article references, study characteristics, species diversity), on which we will perform queries to produce the tables, figures and maps that will compose the systematic map.

**Supplementary Information:**

The online version contains supplementary material available at 10.1186/s13750-022-00280-0.

## Background

Agriculture is the most abundant land use in Europe, covering approximately 45% of the total land area of the EU-27 [1]. The intensification of agricultural practices over the last decades has profoundly modified the functioning of agroecosystems and threatened its biodiversity, resulting in an unfavorable conservation status for 76% of agricultural habitats and 70% of their inhabiting species [2, 3]. The drivers of biodiversity loss are diverse, but the main ones are the simplification and homogenization of the landscape, the loss of semi-natural habitats and the increased application of fertilizers and pesticides on fields [4–8]. The decline of biodiversity in agroecosystems raises considerable concerns about the deficiency of ecosystem services essential for agricultural productivity [9–11], such as pollination, habitat maintenance, formation of soils and pest regulation. The protection of biodiversity and associated ecosystem services is thus a crucial step to ensure the long-term sustainability of farming systems.

Assessing the state and trend of biodiversity in agricultural land is a major challenge, especially given the variety of agricultural management practices (AMPs) and the difficulty of choosing indicator species groups (ISGs) that are ecologically meaningful and representative of biodiversity [8, 12–16]. Currently, only birds and butterflies are monitored in agricultural areas at the European scale, both showing substantial declines over the past decades [17, 18]. There is however numerous other ISGs monitored in national programs or for specific research projects [19–21], and their use depends on the scale considered, the specific context and the objectives of the project [15]. Similarly, while the most common quantitative metric of an ISG diversity is its species richness (number of species), more complex measurements of species heterogeneity (i.e., species evenness, or Shannon index) are often used, these metrics showing sometimes different trends [22, 23]. Consequently, the large number of ISGs, the numerous methods for monitoring them, and the various types of diversity response measured render their utilization and their interpretability more complex, highlighting the pressing need for synthesis in this topic.

**Fig. 1:**
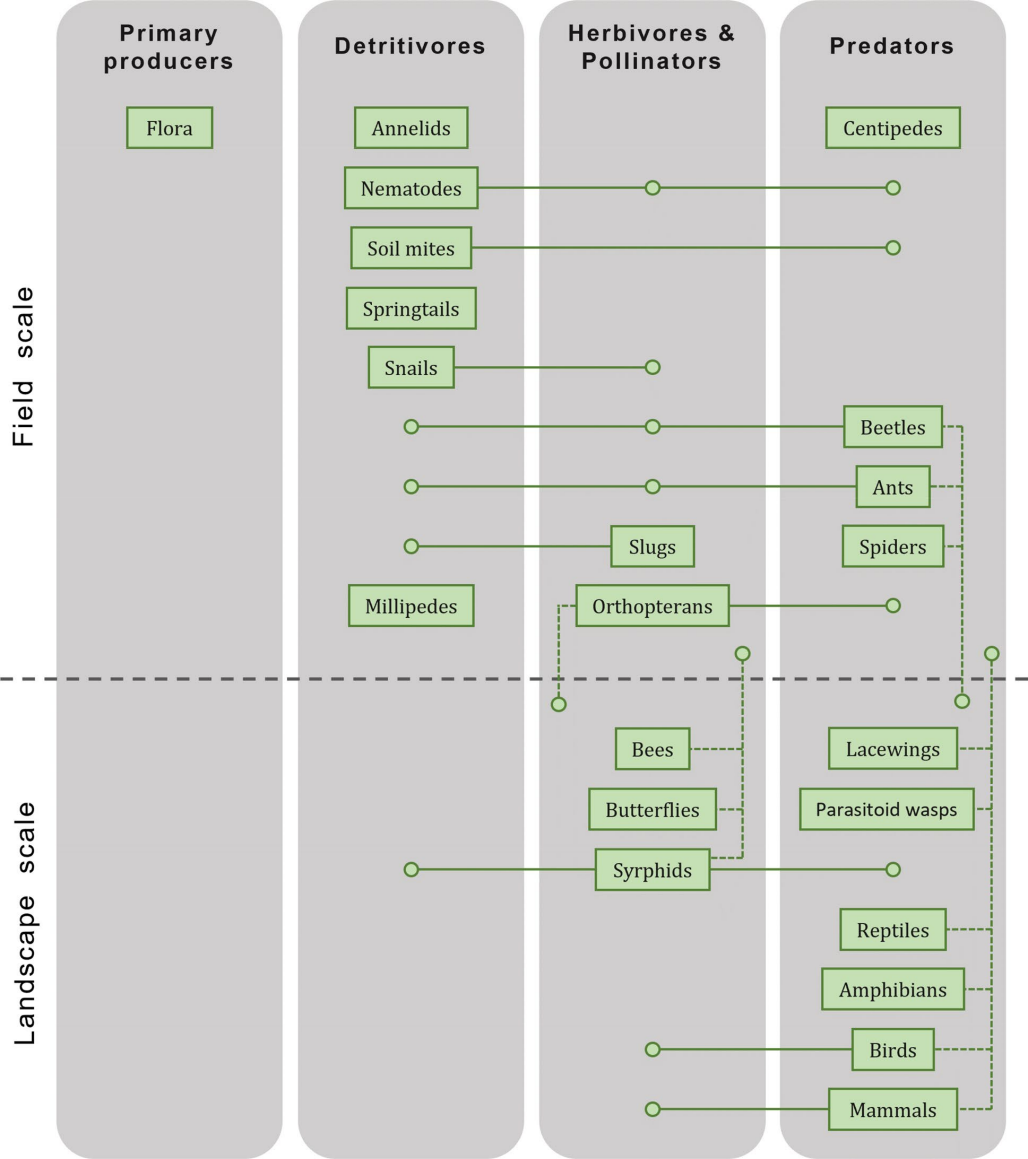
Indicator species groups (ISGs). Representation of the twenty-two ISGs selected for the systematic map based on their trophic level (vertically) and scale of indication (horizontally). Among an ISG, these two parameters might differ between species or stages of development (i.e., larva or adult), so the main classification is indicated by its name in a rectangle, and the other possibilities by a dot connected with a line (plain to indicate possible variations in trophic level and dashed in the scale of indication). These classifications are not exhaustive and are primarily intended to provide an overview of the different ISGs. Adapted from Fig. 6.1 in [19]

The aim of this systematic map is to gather and describe the literature documenting the effects of the main European lowland AMPs on a representative set of ISGs and report them following the systematic evidence synthesis standards (ROSES checklist provided in Additional file 1; see [24] for systematic map specificities). We considered ten main categories of AMPs representative of the diversity of lowland agricultural practices in Europe: soil preparation, fertilization, sowing, irrigation, crop protection, harvesting, mowing, grazing, intermediate cropping, and ecological infrastructure implementation (Additional file 2). We selected the ISGs based on their relevance in biodiversity conservation, ecosystem functionality, use as indicator and provision of ecosystem services, resulting in a set of twenty-two candidates covering a wide range of trophic levels and ecological niches: flora, mammals, birds, reptiles, amphibians, spiders, bees, parasitoid wasps (ichneumonids and braconids), orthopterans, butterflies, beetles (carabids, coccinellids and staphylinids), syrphids, lacewings, ants, slugs, snails, annelids, nematodes, soil mites, springtails, millipedes, and centipedes (Fig. 1; Additional file 3). Our main objectives are threefold: (1) report the effect of the AMPs on the different ISGs, (2) record the different metrics of species diversity used and their possible concordant/antagonistic responses and (3) identify the monitoring methods used, while being particularly attentive to the emergence of novel techniques such as the use of drones or genetic methods.

This systematic map is part of the *Indicate* project [25] aiming at measuring and optimizing the environmental impact of Swiss farms. In this context, we especially focused on the main types of agricultural fields present in the Swiss landscape, grouped in four main categories (annual crops, perennial crops, grasslands, and ecological infrastructures; Additional file 4). Although selected for a Swiss project, the field types considered also correspond to the main European crop types [26], which ensures that the results obtained in this review will also be applicable in the other European countries. The present systematic map will help prioritizing future scientific research by identifying knowledge gaps, as well as providing a synthesis of knowledge for stakeholders in the field and providing tools for decision makers to evolve toward more sustainable agriculture.

## Objective of the review

The goal of this systematic map is to determine the current state of knowledge regarding the effects of the main European lowland AMPs on biodiversity and will be directed towards three main objectives. First, we will report the effects of the AMPs on ISGs covering a wide range of trophic levels and ecological niches. Second, we will record the metrics of species diversity measured and their response trends (possibly concordant/antagonistic responses). Third, we will note the methods used to monitor the ISGs, with a particular interest in the emergence of novel techniques such as the use of unmanned aircraft systems or genetic methods. Combined, these three objectives will allow to assess the current research state on the topic, provide guidance in the selection of ISGs and associated measurement methods, as well as detecting knowledge gaps that could be filled by further research.

### Primary question

What evidence exists on the effect of the main European lowland crop and grassland management practices on biodiversity indicator species groups?

### Components of the primary question

Based on the PICO framework [24], which enables to define a research question based on four main themes (Population, Intervention, Comparator and Outcome), the primary question components are:

- Population (P): The biodiversity indicator species groups (ISGs)
- Intervention (I): The European lowland agricultural management practices (AMPs)
- Comparator (C): The comparison before/after AMP interventions, between AMPs and controls, or between different AMPs
- Outcome (O): Measure of change of the ISGs (i.e., diversity, abundance, or evenness)

### Secondary questions

We have identified five secondary questions that we will address in this systematic map.

1. What are the most surveyed ISGs? Are there spatial or temporal variations?
2. What are the main ISGs monitoring methods? Are there trends towards a change in monitoring methods for some ISGs?
3. What types of diversity measurements (i.e., structural, or functional biodiversity measurements) are most often reported?
4. When a study is reporting multiple diversity measurements, are the results consistent or do they differ?
5. Are the ISGs generally surveyed alone or combined? Which combinations are the most frequent?

## Methods

### Search terms and languages

**Fig. 2:**
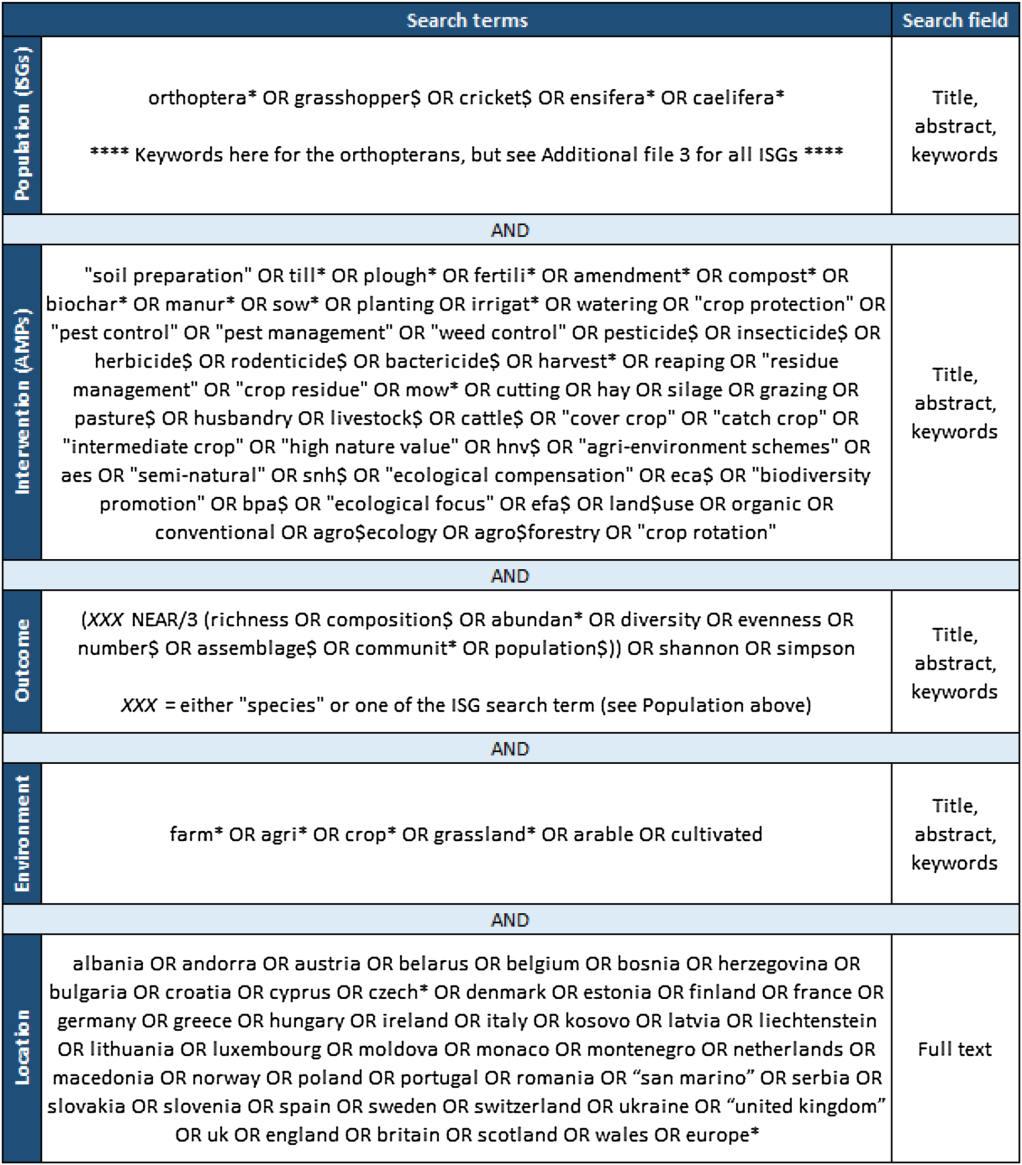
Search string structure. Based on the PICO framework, we defined search terms for the Population, Intervention, and Outcome categories, and completed it with two additional Environment and Location categories to focus on European lowland agricultural environments. The present example illustrates a search on Web of Science for the ISG “orthopterans” (for all ISG search terms, see Additional file 3). Wild card (*) allows to include alternate forms of the word, dollar sign ($) allows to include an additional character, and quotation marks (“”) limit the search to exact wording structure

We relied on different reviews, meta-analyses, books, reports or scientific articles (see for example [8, 13–15, 19, 27–31]) to develop a list of ISGs used in various research domains (nature conservation, ecosystem functionality, biodiversity indicator or ecosystem services). We identified twenty-two ISGs: flora, mammals, birds, reptiles, amphibians, spiders, bees, parasitoid wasps (ichneumonids and the braconids), orthopterans, butterflies, beetles (carabids, coccinellids and staphylinids), syrphids, lacewings, ants, slugs, snails, annelids, nematodes, soil mites, springtails, millipedes, and centipedes. These ISGs cover a wide range of ecological niches and trophic levels (Fig. 1). To collect the literature corresponding to this preliminary list we developed a set of ninety-three search terms (Additional file 3, see *Population* in Fig. 2). We acknowledge that soil microorganisms (i.e., fungi, bacteria, or archaea) are also an important component of biodiversity in agriculture (also in term of ecosystem services), but we finally decided not to include them as ISG due to the too large number of articles that were collected for this group (between 20′000 and 50′000 additional references depending on the keywords).

AMPs in European arable systems are spatially and temporally diverse and it is thus challenging to group them into broad but meaningful categories. Here, we considered ten main AMPs categories: soil preparation, fertilization, sowing, irrigation, crop protection, harvesting, mowing, grazing, intermediate cropping, and ecological infrastructure implementation. We built a set of fifty-five search terms to effectively gather literature corresponding to these main AMPs (Additional file 2; see *Intervention* in Fig. 2).

To record the effects of AMPs on ISGs, we are interested in studies reporting a difference or a change in the diversity, abundance, or survival of the latter. We combined eleven search terms (see *Outcome* in Fig. 2) to include taxonomic, structural, and functional diversity indices. Each of these terms was then associated with the word “species” (i.e., “species richness”) or with one of the ISG search term (i.e., “spider richness”). When the search platform offered the possibility of using proximity operators, they were combined with the Boolean operator “NEAR/3” (in Web of Science) to find records where both terms are within three words of each other (i.e., “richness of spiders”), otherwise they were combined with “AND”.

To restrict the literature search to European agricultural environments, we defined two additional sets of keywords. Six “Environment” keywords aimed at focusing on agricultural landscapes (crop and grassland), and forty-nine “Location” ones restricted the search to European countries (see *Environment* and *Location* in Fig. 2). The geographical range of the study includes most continental Europe (Additional file 5), apart from Russia and Turkey (and countries further east of the latter), and islands as that are commonly known to have different conditions from the continent (i.e., species guilds, types of agriculture or weather conditions).

To account for alternative spelling or hyphenation, search terms were truncated and a wild card (*) added to include alternate forms of the word (i.e., fertili* to account for fertilizer, fertilizers, fertilization, fertilizing, and associated British spelling) or a dollar sign ($) to allow the inclusion of an additional character (i.e., bird$ to account for bird or birds, but not for birding or birdwatch; only available on Web of Science platform). Quotation marks were used to limit the search to exact phrases (i.e., “pest control”). All literature searches will be performed with terms in English.

Finally, as this systematic map is conducted as part of a project aiming at measuring and optimizing the environmental impact of farms in Switzerland (*Indicate* project; [25]), we focused on the type of agricultural fields present in the Swiss lowland agricultural environment (Additional file 6). These fields were grouped in four main categories: annual crops, perennial crops, grasslands, and ecological infrastructures (Additional file 4). They also correspond to the main field types cultivated in Europe (except for olive groves, citrus, nuts, and some fruits [26]), which ensures that the data collected in this study and the conclusions we will be able to draw from it will be useful and applicable to all European countries. We did not define specific keywords to select for field types during the literature search phase, but we will use them as inclusion/exclusion criteria during the screening process (see *Eligibility criteria).*

### Search strings

Search terms within categories will be combined using the Boolean operator “OR”, and between categories using the Boolean operator “AND” (Fig. 2). This implies that studies must include at least one term of each of the five categories to be retained. When possible, the search will be restricted to the article title, abstract and keywords, except for the “Location” search terms that will be screened across the full text. Due to keyword number limitations in the searches, we conducted a literature search separately for each ISG. This means that the keywords for the categories Intervention, Outcome, Environment and Location were combined to each ISG keywords successively (see Fig. 2 for an example).

### Publication databases

We will search for relevant literature on Web of Science Core Collection and CABI platforms.

### Internet searches

We will conduct an internet search on the Google Scholar website using a simplified search string (Additional file 7) at the whole text level (as it is not possible to restrict the search fields in Google Scholar). The first 500 results will be exported in Excel format and screened. To reduce the algorithm biases associated to previous internet searches, browser history and cookies will be disabled during the internet search and the “private” navigation mode used.

### Supplementary searches

A search for grey literature at the European scale would go beyond the scope and resources of this systematic map. So, supplementary searches will be carried out for Switzerland only, to specifically collect relevant literature that has to be considered in the context of the *Indicate* project. We will search for grey literature in English, French, German, and Italian on Swiss specialized websites (Additional file 7). In addition, we will ask for additional Swiss grey literature through the professional networks of the research team. The final analyses will be conducted with and without the grey literature to assess its impact and avoid any grey-literature-based bias in the conclusions.

### Comprehensiveness of the search

To evaluate the comprehensiveness of the literature search, we will compare different search strings results with a test-list of articles considered to be relevant. To produce the test list, we first selected sixty articles that we considered to be appropriate and wanted to obtain in the literature search. Secondly, to ensure the diversity and representativeness of the test-list, we added pertinent literature cited in five key publications on biodiversity in agriculture: a review on the biodiversity in agricultural areas [12], a review of soil biodiversity [14], a European project on agricultural biodiversity [32], and two comprehensive research articles on Swiss biodiversity in agriculture [33, 34]. This resulted in a test-list of ninety articles (Additional file 8), published over a period of thirty years (from 1991 to 2021) in thirty-nine different scientific journals.

### Article screening and study eligibility criteria

#### Screening process

First, as we expect the different search sources to report several times the same references, duplicates will be removed based on the DOI identifier, and on title for references without DOI. Then, the study screening process will successively be performed at the title, abstract and full-text levels. At each level, articles will be classified as relevant (included in the review) or irrelevant (excluded from the review), or uncertain. In the latter case, articles will be passed to the next level of screening and reevaluated (i.e., articles uncertain at the title screening level, will be passed to the abstract screening stage). For each rejected article, we will record the level (title, abstract, full text) and the reason (list of choices) of exclusion, which will be published with the systematic map. Full articles will be collected from the literature access of the Agroscope, the ETH Zürich and the University of Lausanne (UNIL).

To guide reviewers’ choices of including or excluding an article, we defined a set of criteria (see *Eligibility criteria*), and to assess the repeatability of the screening process we will compare the choices made by different reviewers. To do so, a subset of 150 articles will be assessed by two reviewers and their agreement evaluated using a Cohen’s kappa coefficient (k > 0.6 considered as consistent). This will be repeated at each screening level. In case of inconsistency (k < 0.6), the reviewers will discuss to resolve the reasons of inconsistency in their choices, adapt the criteria, and then screen a new subset of articles, until consistency is reached. Even when consistency will be sufficient (k > 0.6), reviewers will discuss and solve the remaining disagreements to ensure a high replicability.

#### Eligibility criteria

To be included in the systematic map, articles must fulfill nine conditions:

- Eligible population: articles must include at least one of the ISGs.
- Eligible intervention: articles must include at least one of the AMPs. One-time events will be excluded (i.e., unique pollution event), as well as studies which do not directly study the effect of AMPs on ISGs (i.e., bird population fluctuation through time in agricultural areas, without specific AMPs associated).
- Eligible comparator: articles must compare ISGs before/after intervention, between an intervention and a control, or between different interventions. Studies reporting ISGs without further comparison will be excluded.
- Eligible outcome: articles must report a measure of species diversity (i.e., richness, abundance, or evenness), otherwise they will be excluded.
- Eligible environment: articles must report research conducted in lowland agricultural landscape. Studies conducted in non-lowland farming landscapes (i.e., forests, or alpine environments) will be discarded.
- Eligible location: articles must report studies conducted on the European mainland. Studies conducted outside the geographical area under consideration will be discarded.
- Eligible study design: articles must report and analyze monitoring or experimental field-data. Modelling papers, books, reviews, or meta-analyses will be excluded.
- Eligible crop types: articles must report the effect of an AMP on an ISG in one of the crop types considered in the present study.
- Eligible language: articles must be written in English.

We developed a list of inclusion/exclusion criteria to guide and standardize the literature screening process (Additional file 9).

#### Study validity assessment

We will not perform a critical appraisal of study validity, but rather extract study characteristics (see *Data coding strategy*) that we will combine to evaluate the relevance of the articles. To do this, we will collect study information (geographical extent, type of design, duration of the study, number of sampling sites, statistical analyses), ISG specifications (ISG focus, ISG monitoring method description, species list, number of diversity measures calculated) and AMP specifications (AMP focus, intervention description). These extracted study characteristics will then be combined to obtain a study validity score, which will represent the relevance of each article based on the goals of the present study. We therefore intend to attribute to each article a value of fit with the study question (external validity measure), rather than a risk of bias evaluation (internal validity).

#### Data coding strategy

The data extracted from the articles will include bibliographic information, study design and characteristics, ISGs and AMPs studied, and effects reported. The data coding strategy will consist in the combination of three tables, linked by a unique article identifier (see Additional file 10 for an overview of the data coding strategy). The first table, “Article References”, will contain the articles’ bibliographic information. The second table, “Study Characteristics”, will contain information about study location and design. The last table, “Species Diversity”, will summarize the effects of AMPs on ISGs measured outcome according to a four-level classification (positive, negative, neutral, or unclear).

To assess the repeatability of the data collection process, we will compare the data extracted by different reviewers on a subset of 50 articles. For each data to be extracted (Additional file 10), we will calculate the reviewer’s agreement as the percentage of fit. Cases of disagreement will be discussed to improve the collection repeatability (i.e., by clarifying the definition of a variable, reformulating the different categories of a variable in the case of multiple choices, or adding additional variables if necessary). We will report in the systematic map the repeatability of each of the extracted variables.

#### Study mapping and presentation

In the systematic map we will provide answers to the primary and secondary questions developed in the present protocol, in the form of descriptive texts supported by tables, figures and maps. As the tables described in the *Data coding strategy* might be further developed during full-text assessment, we will provide all final table structures and contents in the upcoming publication, as well as the final database produced in the frame of this review. To facilitate the use of these files in other research projects, we will provide detailed definitions of each column and coding schemes, as well as R codes [35] used to perform the queries and analyses needed for the review mapping. Detailed information about the articles included/excluded at the different stages of the screening process will be reported, in addition to any eventual modification to the present protocol. Finally, we will discuss strengths and gaps identified, and propose future research to improve our understanding of the effects of the agricultural management practices on biodiversity.

## Supplementary Information

**Additional file 1.**ROSES for Systematic Map Protocols, Version 1.0

**Additional file 2. **Agricultural management practices (AMPs) considered in the systematic map

**Additional file 3. **Indicator species groups (ISGs) included in the systematic map

**Additional file 4. **Crop and grassland field types considered in the systematic map

**Additional file 5. **Geographical range considered in the systematic map

**Additional file 6. **Land-use in Swiss lowland agricultural landscape

**Additional file 7. **Literature search strings

**Additional file 8. **The ninety publications included in the test-list to evaluate the comprehensiveness of the literature search

**Additional file 9. **Inclusion and exclusion criteria for guiding the screening process

**Additional file 10. **Data coding strategy

## Data Availability

All data and materials (references and data extraction tables) will be published open access as additional files.
