# Supplementary material for: What evidence exists on the effect of the main European lowland crop and grassland management practices on biodiversity indicator species groups? A systematic map protocol
Source: Environ Evid. 2022 Aug 25;11:27. doi: 10.1186/s13750-022-00280-0 (PMC11378791; doi:10.1186/s13750-022-00280-0)
Supplement: Supplementary file 1 — Additional file 1.ROSES for Systematic Map Protocols, Version 1.0 [file 13750_2022_280_MOESM1_ESM.docx]

| **ROSES for Systematic Map Protocols. Version 1.0** |
| --- |
| November 2017 |
| Neal R Haddaway, Biljana Macura, Paul Whaley, and Andrew Pullin |
|  |
| **Column descriptions** |
| Section/sub-section - The sections or sub-sections listed below correspond to sections in CEE systematic review and map protocols and final reports: please see the relevant text under these subheadings for information within a specific review. |
| Topic - the section or sub-section is further broken down for the purposes of clarity. |
| Description/Further explanation - Details of what is needed in each section or sub-section are provided, along with practical advice and links to relevant guidance. |
| Checklist/Meta-data - Checklist items MUST be completed. Meta-data items correspond to information that should be reported as data or short descriptions that will be used to populate the Summary Record and Flow Diagram. Any meta-data items where stages were not performed (e.g. grey literature searching), this should be stated (e.g. ‘Not performed’). |
| Author response - authors should select or write a response for meta-data, or select 'yes' or 'no' for checklist items. Further details may be provided in Comments (see below). |
| Comments - authors may supplement their response with a brief comment. |
|  |
| *Please cite as: Haddaway NR, Macura B, Whaley P, and Pullin AS. 2017. ROSES for Systematic Map Protocols. Version 1.0. DOI: 10.6084/m9.figshare.5897284.* |

| **Section / sub-section** | **Topic** | **Description** | **Further explanation** | **Checklist/Meta-data** | **Author response** |
| --- | --- | --- | --- | --- | --- |
| Title | Title | The title must indicate that it is a systematic map protocol, and must indicate if it is an update/amendment: e.g. "A systematic map update protocol...". | The title should normally be the same or very similar to the review question. | Meta-data | What evidence exists on the effect of the main European lowland crop and grassland management practices on biodiversity indicator species groups? A systematic map protocol |
| Type of review | Type of review | Select one of the following types of review: systematic map, systematic map update, systematic map amendment | See CEE Guidance on systematic mapping [1], and on amendments and updates [2] | Meta-data | systematic map |
| Authors contacts | Authors contacts | The full names, institutional addresses, and email addresses for all authors must be provided. |  | Checklist | Yes |
| Abstract | Structured summary | Abstract must not exceed 350 words and must include two sections 1) Background, the context and purpose of the review, including the review question; 2) Methods, how the review will be conducted and the outputs that are expected (specifically mention search strategy, inclusion criteria, critical appraisal, data extraction and synthesis). |  | Checklist | Yes |
| Background | Background | Describe the rationale for the review in the context of what is already known. Protocol must indicate why this study was necessary and what it aims to contribute to the field. | A theory of change and/or conceptual model can be presented that links the intervention or exposure to the outcome. | Checklist | Yes |
| Stakeholder engagement | Stakeholder engagement | The planned/actual role of stakeholders throughout the review process (e.g. in the formulation of the question) must be described and explained (using a broad definition of ‘stakeholder’, including e.g. researchers, funders and other decision-makers; see [3]) |  | Checklist | Yes |
| Objective of the review | Objective | Describe the primary question and secondary questions (when applicable). | The primary question is the main question of the review. Secondary questions are usually linked to sources of heterogeneity (effect modifiers). | Checklist | Yes |
|  | Definitions of the question components | Break down and summarise question key elements e.g. population, intervention(s)/exposure(s), comparator(s), and outcome(s). | For other question types see [4,5] | Meta-data | * Population (P): The biodiversity indicator species groups (ISGs) * Intervention (I): The European lowland agricultural management practices (AMPs) * Comparator (C): The comparison before/after AMP interventions, between AMPs and controls, or between different AMPs  * Outcome (O): Measure of change of the ISGs (i.e., diversity, abundance, or evenness). |
| Methods |  |  |  |  |  |
| Searches | Search strategy |  | Details regarding search strategy testing should be provided. | Checklist | Yes |
|  | Search string | Provide Boolean-style full search string and state the platform for which the string is formatted (e.g. Web of Science format) |  | Meta-data | Provided in Additional file 7 and in Figure 2 |
|  | Languages – bibliographic databases | List languages to be used in bibliographic database searches. |  | Meta-data | English |
|  | Languages – grey literature | List languages to be used in organizational websites searches and web-based search engines. |  | Meta-data | English, French, German, and Italian |
|  | Bibliographic databases | Provide the number of bibliographic databases to be searched. |  | Meta-data | 2 |
|  | Web – based search engines | Provide the number of web – based search engines to be searched. |  | Meta-data | 1 |
|  | Organisational websites | Provide the number of organisational websites to be searched. |  | Meta-data | 6 |
|  | Estimating the comprehensiveness of the search | Describe the process by which the comprehensiveness of the search strategy was assessed (i.e. list of benchmark articles). |  | Checklist | Yes |
|  | Search update | Describe any plans to update the searches during the conduct of the review. | Optional. A search update is good practice if original searches were performed more than two years prior to review completion. | Checklist | Yes |
| Article screening and study inclusion criteria | Screening strategy | Describe the methodology for screening articles/studies for relevance/eligibility. |  | Checklist | Yes |
|  | Consistency checking | Describe clearly the process for checking consistency of decisions including the levels at which consistency checking will be undertaken and estimated proportion of articles/studies that will be screened and checked for consistency by two or more reviewers (e.g. Titles (10%), abstracts (10%), full text (10%)). |  | Checklist | Yes |
|  | Inclusion criteria | Describe the inclusion criteria used to assess relevance of identified articles/studies. These must be broken down into the question key elements (e.g. relevant subject(s), intervention(s)/exposure(s), comparator(s), outcomes, study design(s)) and any other restrictions (e.g. date ranges or languages). |  | Checklist | Yes |
|  | Reasons for exclusion | State that you will provide a list of articles excluded at full text with reasons for exclusion. |  | Checklist | Yes |
| Critical appraisal | Critical appraisal strategy | Describe here the method you propose for critical appraisal of study validity (including assessment of individual studies and the evidence base as a whole). | Optional | Checklist | Yes |
|  | Critical appraisal used in synthesis | Describe how the information from critical appraisal will be used in synthesis. | Optional | Checklist | Yes |
|  | Consistency checking | Describe how repeatability of critical appraisal of study validity will be tested. | Optional | Checklist | Yes |
| Data extraction | Meta-data extraction and coding strategy | Describe the method for meta-data extraction and coding for studies (potentially providing forms/data sheets (ideally piloted), list if variables to be extracted as meta-data and those that will be coded). |  | Checklist | Yes |
| Data synthesis and presentation | Narrative synthesis strategy | Describe methods to be used for narratively synthesising the evidence base in the form of descriptive statistics, tables (including SM database) and figures. | Vote-counting (tallying of studies based on the direction or significance of their findings) must be avoided. May include a summary of the outputs of critical appraisal of the evidence base as a whole (if planned to be performed in SM). | Checklist | Yes |
|  | Knowledge gap and cluster identification strategy | Describe the methods to be used to identify and/or prioritise key knowledge gaps (unrepresented or underrepresented subtopics that warrant further primary research) and knowledge clusters (well-represented subtopics that are amenable to full synthesis via systematic review). |  | Checklist | Yes |
|  | Demonstrating procedural independence | Describe the role of systematic reviewers (who have also authored articles to be considered within the review) in decisions regarding inclusion or critical appraisal of their own work. | Reviewers who have authored articles to be considered within the review should be prevented from unduly influencing inclusion decisions, for example by delegating tasks appropriately. | Checklist | Yes |
| Declarations | Competing interests | Describe of any financial or non-financial competing interests that the review authors may have. |  | Checklist | Yes |
|  |  |  |  |  |  |
|  |  |  |  |  |  |

| **References** |  |  |  |  |  |
| --- | --- | --- | --- | --- | --- |
| [1] James, K.L., Randall, N.P. and Haddaway, N.R., 2016. A methodology for systematic mapping in environmental sciences. Environmental Evidence, 5(1), p.7. | | | | | |
| [2] Bayliss, H.R., Haddaway, N.R., Eales, J., Frampton, G.K. and James, K.L., 2016. Updating and amending systematic reviews and systematic maps in environmental management. Environmental Evidence, 5(1), p.20. | | | | | |
| [3] Haddaway, N.R., Kohl, C., da Silva, N.R., Schiemann, J., Spök, A., Stewart, R., Sweet, J.B. and Wilhelm, R., 2017. A framework for stakeholder engagement during systematic reviews and maps in environmental management. *Environmental Evidence*, *6*(1), p.11. | | | | | |
| [4] Collaboration for Environmental Evidence. 2018. Guidelines and Standards for Evidence synthesis in Environmental Management. Version 5.0. www.environmentalevidence.org/information-for-authors. | | | | | |
| [5] Leeds Institute of Health Sciences. https://medhealth.leeds.ac.uk/info/639/information_specialists/1500/search_concept_tools. Accessed 12/11/2017. | | | | | |
