# Supplementary material for: What evidence exists on the effect of the main European lowland crop and grassland management practices on biodiversity indicator species groups? A systematic map protocol
Source: Environ Evid. 2022 Aug 25;11:27. doi: 10.1186/s13750-022-00280-0 (PMC11378791; doi:10.1186/s13750-022-00280-0)
Supplement: Supplementary file 2 — Additional file 2. Agricultural management practices (AMPs) considered in the systematic map [file 13750_2022_280_MOESM2_ESM.docx]

**Additional file 2: Agricultural management practices (AMPs) considered in the systematic map.** For each of the ten AMP categories, we performed a pre-screen of the literature to collect terms commonly used to describe them (associated terms) and based on those we developed the search terms we will use in the systematic map (search terms, here for Web of Science). Broader search terms were defined and added to the list (additional search terms).

| **AMP category** | **Associated terms (not exhaustive)** | **Search terms** |
| --- | --- | --- |
| Soil preparation | soil preparation, tillage, tilling, plough(ing), conventional / intensive / reduced / zero / traditional tillage. | "soil preparation" OR till* OR plough* |
| Fertilization | fertiliz(s)ation(s), fertiliz(s)er(s), (in)organic fertiliz(s)er(S), soil amendment(s), compost, biochar, manure, manuring | fertili* OR amendment* OR compost* OR biochar* OR manur* |
| Sowing | sowing, sowed, sown crop, planting | sow* OR planting |
| Irrigation | irrigation, irrigate, irrigated, watering | irrigat* OR watering |
| Crop protection | crop protection, pesticide(s), insecticide(s), fungicide(s), herbicide(s), rodenticide(s), bactericide(s), pest control / management, integrated pest management, biological pest control, weed control | "crop protection" OR "pest control" OR "pest management" OR "weed control" OR pesticide$ OR insecticide$ OR herbicide$ OR rodenticide$ OR bactericide$ |
| Harvesting | harvest, harvesting, harvested, harvester, reaping, residue management, crop residue | harvest* OR reaping OR "residue management" OR "crop residue" |
| Mowing | mowing, mown, bar / rotative mower, cutting, hay, silage | mow* OR cutting OR hay OR silage |
| Grazing | grazing, pasture(s), livestock(s), husbandry, cattle(s) | grazing OR pasture$ OR husbandry OR livestock$ OR cattle$ |
| Intermediate crop | cover / catch / intermediate crop | "cover crop" OR "catch crop" OR "intermediate crop" |
| Ecological infrastructures | high nature value (HNV), agri-environment schemes (AES), semi-natural habitats (SNH), biodiversity promotion areas (BPA), ecological focus areas (EFA) | "high nature value" OR hnv$ OR "agri-environment schemes" OR aes OR "semi-natural" OR snh$ OR "ecological compensation" OR eca$ OR "biodiversity promotion" OR bpa$ OR "ecological focus" OR efa$ |
| **Additional search terms (broader scope than the AMPs above)** | | |
| land$use OR organic OR conventional OR agro$ecology OR agro$forestry OR "crop rotation" | | |
