# Supplementary material for: What evidence exists on the effect of the main European lowland crop and grassland management practices on biodiversity indicator species groups? A systematic map protocol
Source: Environ Evid. 2022 Aug 25;11:27. doi: 10.1186/s13750-022-00280-0 (PMC11378791; doi:10.1186/s13750-022-00280-0)
Supplement: Supplementary file 3 — Additional file 3. Indicator species groups (ISGs) included in the systematic map [file 13750_2022_280_MOESM3_ESM.docx]

**Additional file 3: Indicator species groups (ISGs) included in the systematic map.** For each of the twenty-two ISGs, we pre-screened the literature to collect terms commonly used to describe them (associated terms) which were then used to develop the search terms (search terms, for Web of Science and CABI).

| **ISG** | **Associated terms (not exhaustive)** | **Search terms (WOS)** | **Search terms (CABI)** |
| --- | --- | --- | --- |
| Amphibians | amphibia, amphibian(s), frog(s), toad(s), salamander(s), newt(s), anura, anuran(s), caudata, urodela | amphibia* OR frog$ OR toad$ OR salamander$ OR newt$ OR anura* OR caudata OR urodela | amphibia* OR frog OR frogs OR toad OR toads OR salamander OR salamanders OR newt OR newts OR anura* OR caudata OR urodela |
| Annelids | annelid(s), annelida, annelidan(s), earthworm(s), oligochaeta, lumbricidae | oligochaeta OR annelid* OR lumbricidae OR earthworm$ | oligochaeta OR annelid* OR lumbricidae OR earthworm OR earthworms |
| Ants | ant(s), formicidae | formicidae OR ant OR ants | formicidae OR ant OR ants |
| Bees | apoidae, bee(s), bumblebee(s), wild bee(s), pollinator(s) | apoidea OR bee OR bees OR bumblebee$ | apoidea OR bee OR bees OR bumblebee OR bumblebees |
| Beetles (carabids, coccinellids, and staphylinids) | coleoptera, coleopteran(s), carabid(s), carabidae, carabid beetle(s), ground beetle(s), coccinellid(s), coccinellidae, ladybird beetle(s), staphylinid(s), staphylinidae, staphilinid beetle(s), rove beetle(s) | carabid* OR beetle$ OR coccinellid* OR ladybird* OR staphylinid* | carabid* OR beetle OR beetles OR coccinellid* OR ladybird* OR staphylinid* |
| Birds | bird(s), aves, avian, neognath(s), neognathae | aves OR neogonath* OR bird$ | aves OR neogonath* OR bird OR birds |
| Butterflies | butterfly, butterflies, lepidoptera, lepidoteran(s), rhopalocera, papilionoidea, Hesperiidae/skippers, Papilionidae, Pieridae, Lycaenidae, Riodinidae, Nymphalidae | butterfl* OR lepidoptera* OR rhopalocera* OR hesperi* OR papilion* OR pierid* OR lycaenid* OR riodinid* OR nymphalid* | butterfl* OR lepidoptera* OR rhopalocera* OR hesperi* OR papilion* OR pierid* OR lycaenid* OR riodinid* OR nymphalid* |
| Centipedes | centipede(s), chilopoda, chilopod(s), myriapoda, myriapod(s) | myriapod$ OR chilopod$ OR centipede$ | myriapod* OR chilopod* OR centipede OR centipedes |
| Flora | flora, floristic, flower(s), weed(s), flowering plant(s), angiospermae, angiosperm(s), botanic(al) | flora OR floristic OR flower* OR weed$ OR botanic* | flora OR floristic OR flower* OR weed* OR botanic* |
| Lacewings | chrysopidae, chrysopid(s), common lacewing(s), green lacewing(s), neuroptera, neuropteran(s) | chrysopid* OR lacewing$ | chrysopid* OR lacewing OR lacewings |
| Land slugs | slug(s), land slug(s), gastropod(s), gastropoda, mollusc(s), mollusk(s) | gastropod* OR mollus* OR slug$ | gastropod* OR mollus* OR slug OR slugs |
| Land snails | snail(s), land snail(s), gastropod(s), gastropoda, mollusc(s), mollusk(s) | gastropod* OR mollus* OR snail$ | gastropod* OR mollus* OR snail OR snails |
| Mammals | mammal(s), mammalia, mammalian(s), bat(s), chrioptera, chiropteran(s), rodentia, rodent(s), mouse/mice, vole(s), lagomorph(s), lagomorpha, hare(s), rabbit(s), carnivora, carnivoran(s), fox(es), ungulate(s), artiodactyla, artiodactyl(s), deer(s), boar(s), eulipotyphla, hedgehog(s), shrew(s), mole(s) | mammal* OR chiroptera* OR bat$ OR rodent$ OR mouse OR mice OR vole$ OR lagomorph$ OR hare$ OR rabbit$ OR eulipotyphla* OR hedgehog$ OR shrew$ OR mole$ OR carnivora* OR artiodactyl* OR ungulate$ | mammal* OR chiroptera* OR bat OR bats OR rodent OR rodents OR rodentia OR mouse OR mice OR vole OR voles OR lagomorph OR lagomorphs OR lagormorpha OR hare OR hares OR rabbit OR rabbits OR eulipotyphla* OR hedgehog OR hedgehogs OR shrew OR shrews OR mole OR moles OR carnivora* OR artiodactyl* OR ungulate OR ungulates |
| Millipedes | millipede(s), diplopoda, diplopod(s), myriapoda myriapod(s) | myriapod$ OR diplopod$ OR millipede$ | myriapod* OR diplopod* OR millipede OR millipedes |
| Nematodes | nematode(s), nematoda, worm(s), roundworm(s) | nematod* OR roundworm$ | nematod* OR roundworm OR roundworms |
| Orthopterans | orthoptera, orthopteran(s), grasshopper(s), ensifera, ensiferan(s), caelifera, caeliferan(s), cricket(s) | orthoptera* OR grasshopper$ OR cricket$ OR ensifera* OR caelifera* | orthoptera* OR grasshopper OR grasshoppers OR cricket OR crickets OR ensifera* OR caelifera* |
| Parasitoid wasps (ichneumonids and braconids) | ichneumonid(s), ichneumon wasp(s), darwin wasp(s), ichneumonidae, parasitic wasp(s), hymenoptera, hymenopteran(s), ichneumonoidea, braconid(s), braconidae | ichneumon* OR braconid* OR wasp$ | ichneumon* OR braconid* OR wasp OR wasps |
| Reptiles | reptile(s), reptilia(n), snake(s), lizard(s), squamate(s), squamata | reptil* OR squamat* OR snake$ OR lizard$ | reptil* OR squamat* OR snake OR snakes OR lizard OR lizards |
| Soil mites | mite(s), acari, arachnid(s), arachnida, acariformes, parasitiformes | arachnid* OR mite$ OR acari* | arachnid* OR mite OR mites OR acari* |
| Spiders | spider(s), arachnid(s), arachnida, araneae, araneomorphae, araneomorph(s), labidognatha | arane* OR arachnid* OR spider$ | arane* OR arachnid* OR spider OR spiders |
| Springtails | collembola, collembolan(s), springtail(s), hexapod(s) | collembola* OR springtail* OR hexapod* | collembola* OR springtail* OR hexapod* |
| Syrphids | hoverfly, hoverflies, hover flies, syrphid flies, syrphid(s), syrphoidea, syrphidae, diptera | syrphid* OR hover$fl* | syrphid* OR hover*fl* |
