# Supplementary material for: What evidence exists on the effect of the main European lowland crop and grassland management practices on biodiversity indicator species groups? A systematic map protocol
Source: Environ Evid. 2022 Aug 25;11:27. doi: 10.1186/s13750-022-00280-0 (PMC11378791; doi:10.1186/s13750-022-00280-0)
Supplement: Supplementary file 4 — Additional file 4. Crop and grassland field types considered in the systematic map [file 13750_2022_280_MOESM4_ESM.docx]

**Additional file 4: Crop and grassland field types considered in the systematic map.** Fourteen agricultural field types (“Type II”), grouped in four main categories (“Type I”), have been selected for this systematic map (see Additional file 2). The table below provides information about the field types included and excluded for each of the categories.

| **Type I** | **Type II** | **Crop types included** | **Crop types excluded** |
| --- | --- | --- | --- |
| Annual crops | Cereals | wheat, barley, rye, oat, spelt, and triticale | i.e., sorghum, quinoa, millet, buckwheat, or rice |
|  | Maize | maize/corn |  |
|  | Row crops | potato and beet | i.e., sweet potato |
|  | Oleaginous | rapeseed, sunflower, and soybean | i.e., pumpkin oil |
|  | Vegetables | i.e., carrot, onion, lettuce, broccoli, or cauliflower | i.e., tomato, pumpkin, beans, asparagus, melons, berries, aromatic, or medicinal herbs |
|  | Cover crops | i.e., protein pea, faba bean, vetch, alfalfa, clover, lupin, ryegrass, or oat |  |
| Perennial crops | Vineyards | vineyard |  |
|  | Orchards | apple, pear, apricot, cherries, and prune | i.e., peach, nectarine, olive, citrus (i.e., orange, satsuma, or lemon), nuts, kiwis, figs, or avocado |
| Grasslands | Meadows | intensive meadow |  |
|  | Pastures | intensive pasture |  |
| Ecological infrastructures | Grasslands | extensive meadow, extensive pasture, woodland pasture, and litter field |  |
|  | Croplands | wildflower strip, fallow, crop margin, and flower bed in crop |  |
|  | Woody crops | high stem orchard, single tree, tree alley, hedge, bosk, and grove |  |
|  | Special structures | pond, wet ditch, ruderal area, stone heap, and dry wall |  |
