# Supplementary material for: What evidence exists on the effect of the main European lowland crop and grassland management practices on biodiversity indicator species groups? A systematic map protocol
Source: Environ Evid. 2022 Aug 25;11:27. doi: 10.1186/s13750-022-00280-0 (PMC11378791; doi:10.1186/s13750-022-00280-0)

**Additional file 5: Geographical range considered in the systematic map.** The mainland European countries considered are (in alphabetical order) : Albania, Andorra, Austria, Belarus, Belgium, Bosnia, Herzegovina, Bulgaria, Croatia, Cyprus, Czechia, Denmark, Estonia, Finland, France, Germany, Greece, Hungary, Ireland, Italy, Kosovo, Latvia, Liechtenstein, Lithuania, Luxembourg, Moldova, Monaco, Montenegro, Netherlands, Macedonia, Norway, Poland, Portugal, Romania, San Marino, Serbia, Slovakia, Slovenia, Spain, Sweden, Switzerland, Ukraine, United Kingdom (UK), England, Britain, Scotland and Wales. Islands are excluded from the scope of this study. The islands were excluded as they are commonly known to have different conditions from the continent (i.e., species guilds, types of agriculture or weather conditions).


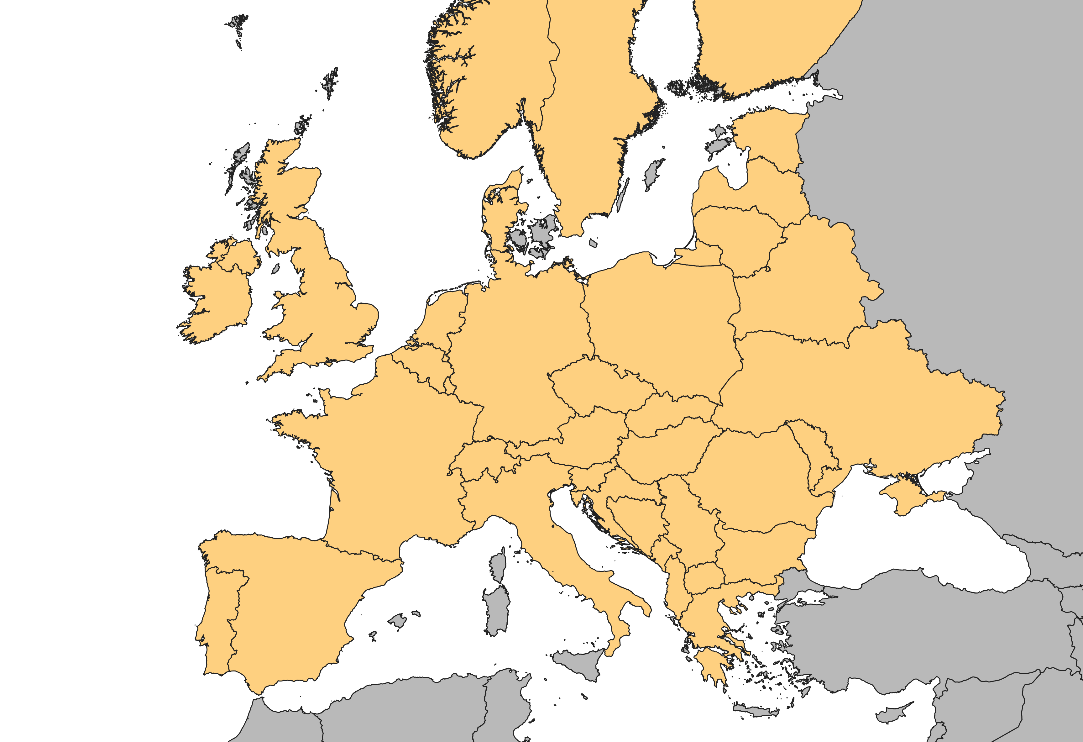

Supplement: Supplementary file 5 — Additional file 5. Geographical range considered in the systematic map [file 13750_2022_280_MOESM5_ESM.docx]
