# Supplementary material for: What evidence exists on the effect of the main European lowland crop and grassland management practices on biodiversity indicator species groups? A systematic map protocol
Source: Environ Evid. 2022 Aug 25;11:27. doi: 10.1186/s13750-022-00280-0 (PMC11378791; doi:10.1186/s13750-022-00280-0)
Supplement: Supplementary file 6 — Additional file 6. Land-use in Swiss lowland agricultural landscape [file 13750_2022_280_MOESM6_ESM.docx]

**Additional file 6: Land-use in Swiss lowland agricultural landscape.** The abundance of the different field types in Swiss lowland agricultural areas was obtained from the website of the Federal Statistical Office ([link](https://www.pxweb.bfs.admin.ch/pxweb/fr/px-x-0702000000_106/px-x-0702000000_106/px-x-0702000000_106.px)). Data from the last five years (2017-2021) were averaged and grouped per categories depending on the type of land-use. The land-use categories are ordered according to their abundance (surface in ha). Categories covering less than 0.5% of the agricultural area were considered poorly representative of Swiss crop types and excluded from the analyses (in red).

| **Land-use category** | **Field types included** | **Surface [ha]** | **Surface [%]** |
| --- | --- | --- | --- |
| Grasslands | intensive grassland | 160027,1 | 32,59 |
| Cereals | i.e., wheat, barley, rye, or oat | 101103,9 | 20,59 |
| Maize | maize | 51097,0 | 10,41 |
| Biodiversity structures | meadows, wildflower strips and hedges | 50153,4 | 10,21 |
| Pastures | intensive pastures | 33747,2 | 6,87 |
| Row crops | potatoes and beetroots | 27766,0 | 5,65 |
| Oleaginous | rapeseed, sunflower, and soybeans | 26685,3 | 5,43 |
| Vegetables | i.e., carrots, onions, or lettuce | 13123,1 | 2,67 |
| Vineyards | vineyards | 10659,4 | 2,17 |
| Orchards | i.e., apple, pear, or prunes | 5987,4 | 1,22 |
| Leguminous | i.e., protein peas or faba beans | 4708,5 | 0,96 |
| Others | unspecified or uncategorisable (i.e., hop) | 2381,7 | 0,49 |
| Plant Nurseries | i.e., ornamental or Christmas trees | 1493,2 | 0,30 |
| Berries | i.e., strawberries or raspberries | 970,0 | 0,20 |
| Raw materials | i.e., hemp, flax, rape, or sunflower | 529,8 | 0,11 |
| Tobacco | tobacco | 431,4 | 0,09 |
| Herbs | aromatic and medicinal plants | 172,8 | 0,04 |
