# Supplementary material for: What evidence exists on the effect of the main European lowland crop and grassland management practices on biodiversity indicator species groups? A systematic map protocol
Source: Environ Evid. 2022 Aug 25;11:27. doi: 10.1186/s13750-022-00280-0 (PMC11378791; doi:10.1186/s13750-022-00280-0)
Supplement: Supplementary file 7 — Additional file 7. Literature search strings [file 13750_2022_280_MOESM7_ESM.docx]

**Additional file 7: Literature search strings.** We will search for literature on Web of Science Core Collection, CABI, Google Scholar and specialized websites. The tables below provide the search strings we will use in each of them, as well as the different restrictions and the number of references retrieved.

*Web of Science Core collection*

| **Search string** | **Remarks & restrictions** |  |
| --- | --- | --- |
| TI/AB/AK=(*ISG keywords*) **AND**  TI/AB/AK=(((*ISG keywords*) NEAR/3 (richness OR composition$ OR abundan* OR diversity OR evenness OR number$ OR assemblage$ OR communit* OR population$)) OR (species NEAR/3 (richness OR composition$ OR abundan* OR diversity OR evenness OR number$ OR assemblage$ OR communit* OR population$)) OR shannon OR simpson) **AND** TI/AB/AK=("soil preparation" OR till* OR plough* OR fertili* OR amendment* OR compost* OR biochar* OR manur* OR sow* OR planting OR irrigat* OR watering OR "crop protection" OR "pest control" OR "pest management" OR "weed control" OR pesticide$ OR insecticide$ OR herbicide$ OR rodenticide$ OR bactericide$ OR harvest* OR reaping OR "residue management" OR "crop residue" OR mow* OR cutting OR hay OR silage OR grazing OR pasture$ OR husbandry OR livestock$ OR cattle$ OR "cover crop" OR "catch crop" OR "intermediate crop" OR "high nature value" OR hnv$ OR "agri-environment schemes" OR aes OR "semi-natural" OR snh$ OR "ecological compensation" OR eca$ OR "biodiversity promotion" OR bpa$ OR "ecological focus" OR efa$ OR land$use OR organic OR conventional OR agro$ecology OR agro$forestry OR "crop rotation") **AND** TI/AB/AK=(farm* OR agri* OR crop* OR grassland* OR arable OR cultivated) **AND**  ALL=(Albania OR Andorra OR Austria OR Belarus OR Belgium OR Bosnia OR Herzegovina OR Bulgaria OR Croatia OR Cyprus OR Czech* OR Denmark OR Estonia OR Finland OR France OR Germany OR Greece OR Hungary OR Ireland OR Italy OR Kosovo OR Latvia OR Liechtenstein OR Lithuania OR Luxembourg OR Moldova OR Monaco OR Montenegro OR Netherlands OR Macedonia OR Norway OR Poland OR Portugal OR Romania OR “San Marino” OR Serbia OR Slovakia OR Slovenia OR Spain OR Sweden OR Switzerland OR Ukraine OR “United Kingdom” OR “UK” OR England OR Britain OR Scotland OR Wales OR Europe*) | * "*ISG keywords*" corresponds to the keywords developed for each ISG, all provided in Additional file 3.  * "TI/AB/AK" indicates here that the search will be performed at the title (TI), abstract (AB) and keywords (AK) levels. "ALL" indicates that it will be searched at the full-text level.   * Web of Science Core Collection  * No date limitation (the whole database will be screened)  * All languages (non-English articles will be excluded at a later stage)  * All document types (will be selected during the screening).   * All categories (no preselection, but could be restricted in a future step) |  |
|  |  |  |

*CABI*

| **Search string** | **Remarks & restrictions** |  |
| --- | --- | --- |
| et/ab/de:(*ISG keywords*) **AND**  et:(richness OR composition* OR abundan* OR diversity OR evenness OR number* OR assemblage* OR communit* OR population* OR shannon OR simpson) **AND** et/ab/de:("soil preparation" OR till* OR plough* OR fertili* OR amendment* OR compost* OR biochar* OR manur* OR sow* OR planting OR irrigat* OR watering OR "crop protection" OR "pest control" OR "pest management" OR "weed control" OR pesticide* OR insecticide* OR herbicide* OR rodenticide* OR bactericide* OR harvest* OR reaping OR "residue management" OR "crop residue" OR mow* OR cutting OR hay OR silage OR grazing OR pasture* OR husbandry OR livestock* OR cattle* OR "cover crop" OR "catch crop" OR "intermediate crop" OR "high nature value" OR hnv* OR "agri-environment schemes" OR aes OR "semi-natural" OR snh* OR "ecological compensation" OR eca* OR "biodiversity promotion" OR bpa* OR "ecological focus" OR efa* OR land*use OR organic OR conventional OR agro*ecology OR agro*forestry OR "crop rotation")  **AND** et/ab/de:(farm* OR agri* OR crop* OR grassland* OR arable OR cultivated)  **AND**  (Albania OR Andorra OR Austria OR Belarus OR Belgium OR Bosnia OR Herzegovina OR Bulgaria OR Croatia OR Cyprus OR Czech* OR Denmark OR Estonia OR Finland OR France OR Germany OR Greece OR Hungary OR Ireland OR Italy OR Kosovo OR Latvia OR Liechtenstein OR Lithuania OR Luxembourg OR Moldova OR Monaco OR Montenegro OR Netherlands OR Macedonia OR Norway OR Poland OR Portugal OR Romania OR “San Marino” OR Serbia OR Slovakia OR Slovenia OR Spain OR Sweden OR Switzerland OR Ukraine OR “United Kingdom” OR “UK” OR England OR Britain OR Scotland OR Wales OR Europe*) | * "*ISG keywords*" corresponds to the keywords developed for each ISG, all provided in Additional file 3.  * "et/ab/de" indicates here that the search will be performed at the title (et), abstract (ab) and keywords (de) levels. No indication means that it will be searched at the full-text level.   * No date limitation (the whole database will be screened)  * All languages (non-English articles will be excluded at a later stage)  * All document types (will be selected during the screening).   * All categories (no preselection, but could be restricted in a future step)  * All locations (no preselection, but could be restricted in a future step) |  |
|  |  |  |

*Google Scholar*

| **Search string** | **Remarks & restrictions** |
| --- | --- |
| species AND (diversity OR richness OR number OR abundance) AND agriculture AND farming AND europe | * Search at full-text level  * No date limitation  * All languages |

Specialized websites

| **Organization** | **Website** | **Remarks & restrictions** |
| --- | --- | --- |
| Agroscope | [www.agroscope.admin.ch](http://www.agroscope.admin.ch/) | * Languages: English, French, German, Italian  * Additional websites might be added during the literature search and screening phases  * Search strings will be provided in the systematic map publication. |
| FiBL | [www.fibl.org](http://www.fibl.org/) |  |
| Federal Office for Agriculture | [www.blw.admin.ch](http://www.blw.admin.ch/) |  |
| Federal Office for the Environment | [www.bafu.admin.ch](http://www.bafu.admin.ch/) |  |
| Swiss Academy of Sciences | [www.scnat.ch](http://www.scnat.ch/) |  |
| Swiss ornithological institute | [www.vogelwarte.ch](http://www.vogelwarte.ch/) |  |
