# Supplementary material for: What evidence exists on the effect of the main European lowland crop and grassland management practices on biodiversity indicator species groups? A systematic map protocol
Source: Environ Evid. 2022 Aug 25;11:27. doi: 10.1186/s13750-022-00280-0 (PMC11378791; doi:10.1186/s13750-022-00280-0)

**Additional file 9: Inclusion and exclusion criteria for guiding the screening process.** We defined criteria to guide and support the reviewer choice of including/excluding references at each level of the screening process (title, abstract, and full text).


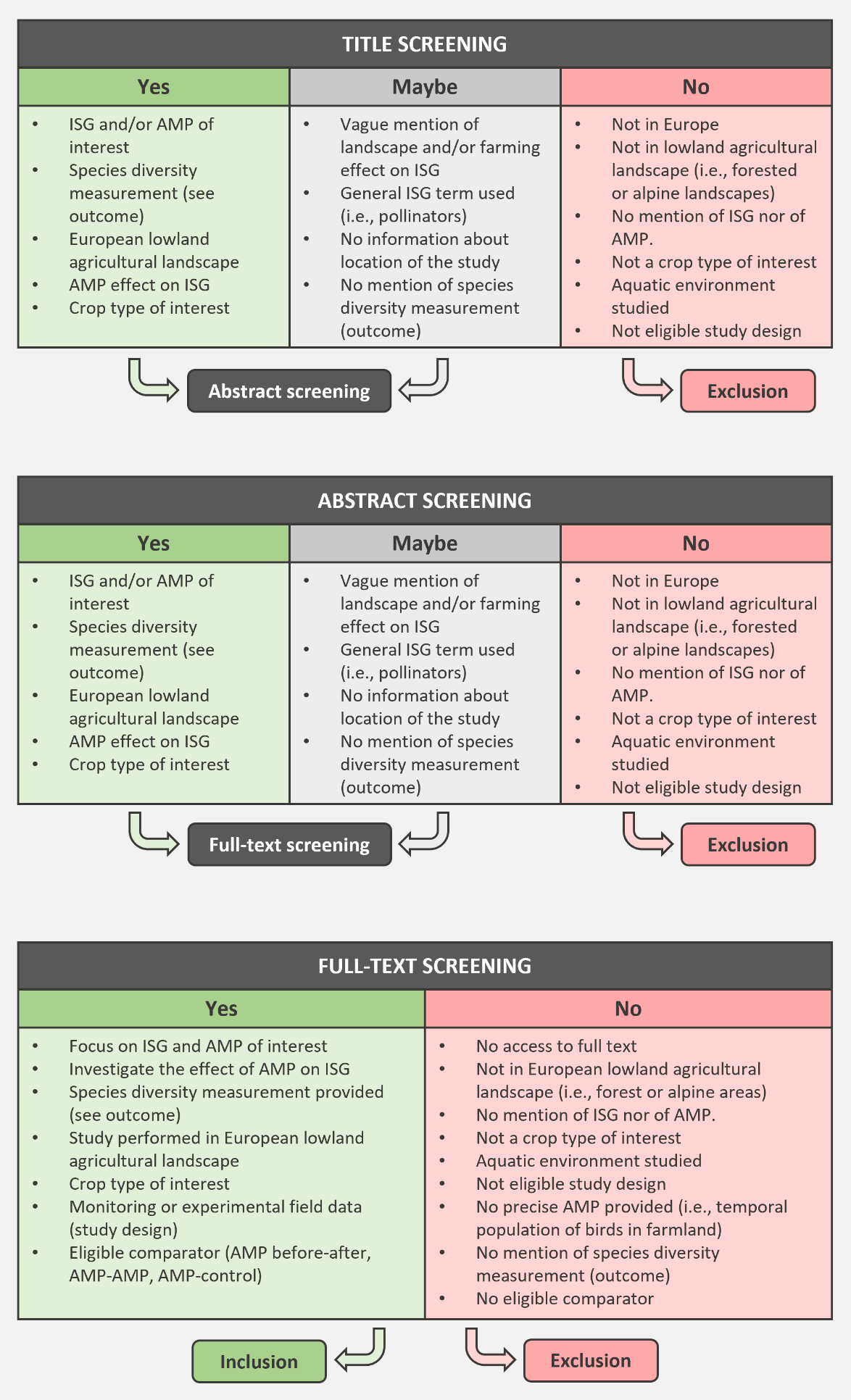

Supplement: Supplementary file 9 — Additional file 9. Inclusion and exclusion criteria for guiding the screening process [file 13750_2022_280_MOESM9_ESM.docx]
